# Anterior Lens Curvature Matters in the Course of Primary Angle Closure: An Analysis Based on Ultrasound Biomicroscopic Imaging

**DOI:** 10.1155/2022/5570633

**Published:** 2022-01-29

**Authors:** Tingting Liu, Mengwei Li, Xiaoxiao Chen, Zhenying Jiang, Xiangmei Li, Xinghuai Sun, Jiajian Wang, Yuhong Chen

**Affiliations:** ^1^Department of Ophthalmology and Visual Science, Eye and ENT Hospital, Shanghai Medical College, Fudan University, Shanghai 200031, China; ^2^NHC Key Laboratory of Myopia, Chinese Academy of Medical Sciences and Shanghai Key Laboratory of Visual Impairment and Restoration (Fudan University), Shanghai 200031, China; ^3^State Key Laboratory of Medical Neurobiology and MOE Frontiers Center for Brain Science, Institutes of Brain Science, Fudan University, Shanghai 200032, China

## Abstract

**Purpose:**

To evaluate the effect of anterior lens curvature in primary angle closure (PAC) and find additional anatomical features of crystalline lens that may predispose primary angle closure to the acute course.

**Methods:**

435 eyes (263 subjects) were enrolled in this study. Four groups of eyes were included based on angle configurations and clinical features: (i) acute primary angle closure (APAC, 140 eyes); (ii) chronic primary angle closure (CPAC, 116 eyes); (iii) primary angle closure suspect (PACS, 84 eyes); and (iv) normal controls (95 eyes). All patients underwent thorough ophthalmic exams including applanation tonometry, gonioscopy, low-coherence interferometry, and ultrasound biomicroscopic imaging. Based on the panoramic anterior segment images from ultrasound biomicroscopic imaging measurements, the radius of anterior lens curvature (ALR) was calculated using the least-squares curve fitting technique. ALR, in addition to axial length (AL), anterior chamber depth (ACD), and lens thickness (LT), was compared among different groups using univariate and multivariate analysis with mixed effects linear model.

**Results:**

APAC, CPAC, and PACS groups all had steeper ALR, shorter AL, shallower ACD, and thicker LT than normal control group. ACD and LT further differ between APAC and CPAC or PACS eyes. Moreover, a steeper ALR was also found in the APAC group as compared to CPAC, PACS, and normal control groups.

**Conclusions:**

A steeper ALR may predispose the acute attack of PAC. In addition to the relative lens position and size, lens curvature is another variable that contributes to the pathophysiological mechanisms of primary angle closure.

## 1. Introduction

Glaucoma is a group of diseases with characteristic optic nerve damage and is the leading cause of irreversible blindness in the world. Primary open angle glaucoma (POAG) and primary angle closure glaucoma (PACG) are the two most common forms of glaucoma, and PACG is more prevalent in China and other Asian countries, causing a more significant healthcare issue in these areas [1]. As compared to POAG, PACG is often more visually destructive and has higher probability of progression to blindness. It is estimated that PACG accounts for about half of the world's glaucoma-related blindness, and about 90% are east Asian [2].

PACG is characterized by obstruction of the drainage pathway, causing intraocular pressure (IOP) rise and ultimately glaucomatous optic neuropathy. The clinical course of PACG can be complex and usually divided into two typical manifestations, i.e. the acute form and the chronic form. The acute angle closure glaucoma (APACG) usually has more severe impact on the visual function that requires emergent management. The dramatic rise of IOP in APACG can not only cause glaucomatous neuropathy but may also cause ischemic neuropathy if not resolved promptly [3, 4].

Since PACG is potentially preventable compared to POAG, identifying relevant risk factors is the key for early detection and prevention. Various anatomical features of anterior segment structure have been found to be related to the development and progression of different clinical course of PACG. Identifying risk factors that may predispose the acute course has great clinical significance. Previous studies have made comparisons between APACG and controls [5], APACG and CPACG [5–7], APACG and glaucomatous eyes with narrow angle [8], APACG and unaffected fellow eyes [7, 9–13], APACG and primary angle closure suspect (PACS) [6, 9, 10, 14] and fellow eyes between APACG and CPACG [15]. Existing predisposing factors for APACG include biometric parameters of anterior chamber (even crowder anterior chamber), angle configuration (even narrower angle), biometric parameters of iris (even thicker and curved iris), and biometric parameters of lens (more anteriorly positioned and thicker), although some of the factors showed discrepancies between studies. Progression analysis studies with PACS suggested that certain anterior segment features including angle opening distance and iris curvature can also have predictive value of disease progression [16, 17].

The crystalline lens plays an important role in PAC and PACG [18]. Relative position and the thickness of lens have been found to be relevant in the pathogenesis of PACG, either through pupillary block mechanism or direct crowding of the anterior angle. Another anatomical feature that may be relevant is the radius of anterior lens curvature (ALR). Recent study using CASIA 2 Optical Coherence Tomography (Tomey, Nagoya, Japan) made comparisons of ALR among PAC/PACG, PACS, and normal controls and found steeper ALR in PAC/PACG, PACS as compared to controls [19]. Whether ALR also plays a role in the onset forms of PACG is not clear. This study focused on the anterior lens curvature and included APAC, CPAC, PACS, and normal eyes for comparison. We used ultrasound biomicroscopic imaging (UBM) to measure ALR. UBM is broadly applied in visualizing angle structure, ciliary body, iris, and lens. The advantages of UBM over other optical instruments such as anterior segment OCT, include a deeper signal penetration, the ability to view zonules and ciliary body at the same time, and an absence of the optical distortions [20].

## 2. Materials and Methods

This hospital-based cross-sectional observational study was conducted at the Eye and ENT hospital of Fudan University and approved by the institutional review board of the Eye and ENT hospital of Fudan University. The study was conducted in adherence to the tenets of the Declaration of Helsinki and written informed consents were obtained from all the subjects.

Patients with histories of PAC or occludable angles were recruited from the glaucoma clinics of Eye and ENT hospital of Fudan University. Patients with open angle under nonindention gonioscopy were recruited as controls in the outpatient clinic. Exclusion criteria included visual acuity lower than 20/200, histories of intraocular surgeries such as trabeculectomy or cataract removal, and secondary angle closures such as explicit histories of ocular trauma, uveitis, or ischemic retinal vascular diseases. Patients with an interocular difference of ACD greater than 1 mm were also excluded in case of an implicit cause of secondary angle closures such as unnoticed history of ocular trauma. The lens opacity was graded using the Lens Opacities Classification System (LOCS) III standards [21]. Eyes with nuclear opalescence, nuclear color, or cortical gradings greater than 3 were excluded to avoid confounding effects of lens opacity.

All patients underwent thorough exams clinically including assessment of visual acuity, slit-lamp biomicroscopy, stereoscopic evaluation of the optic disk using a 90-diopter lens (Volk Optical, Inc., Mentor, OH, USA), intraocular pressure (IOP) measurement with Goldmann applanation tonometry (Haag-Streit, Koniz, Switzerland), and automated white on white perimetry (Humphrey Visual Field Analyzer; Carl Zeiss Meditec, Dublin, CA; and Octopus Automated Perimeter; Haag-Streit, Koniz, Switzerland). Low-coherence interferometry (LenStar 900; Haag-Streit, Koeniz, Switzerland) was used to measure the axial length (AL), central corneal thickness (CCT), anterior chamber depth (ACD), lens thickness (LT), flat keratometry (Kf), and steep keratometry (Ks). Gonioscopy was performed at the time of recruitment by an experienced examiner (YHC) using a 4-mirror lens (Volk Optical, Inc., Mentor, OH, USA). Nonindentation gonioscopy was performed in dim illumination using a shortened slit beam with eyes in the primary gaze position. Indentation gonioscopy was also performed to identify presence of peripheral anterior synechiae (PAS).

### 2.1. Definitions

Four groups were defined based on the following criteria:

1. APAC: eyes with histories of an acute attack of angle closure with at least two of the symptoms of ocular or periocular pain, severe headache, vision-associated nausea/vomiting, decreased vision, and rainbow-colored halos around light and presence of IOP elevation no less than 22 mmHg. Conjunctival injection, corneal epithelial edema, and mid-dilated nonreacting pupil with shallow peripheral anterior chamber were evident if presented during acute phase. All patients had resolved from acute attack with topical medications and sometimes hyperosmotic agents before data collection.
2. CPAC: eyes with occludable angles (at least 180° of the posterior pigmented trabecular meshwork was not visible on nonindentation gonioscopy) and presence of PAS or elevated IOP (>21 mm Hg), with or without evidence of glaucomatous optic neuropathy. No histories or signs of an acute attack (pupil change, iris atrophy or dyspigmentation, glaukomflecken) were present in this group.
3. PACS: eyes with occludable angles (at least 180° of the posterior pigmented trabecular meshwork was not visible on nonindentation gonioscopy, but no signs of trabecular damage such as PAS on indentation gonioscopy), normal IOP (no higher than 21 mmHg), and healthy optic disc.
4. Normal Controls (NC): eyes with open angles on nonindentation gonioscopy, normal IOP (no higher than 21 mmHg), and healthy optic disc.

### 2.2. UBM Imaging and Analysis

**Figure 1:**
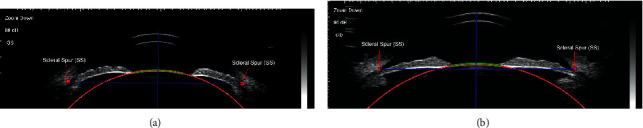
Fitted circle overlapped with the anterior lens surface on an APAC eye (a) and a normal eye (b) using the least-squares curve fitting method. Arrows point to the scleral spurs. The two imaginary blue lines help determine the center of anterior lens surface. The green curve is the 3 mm length of anterior lens surface for curve fitting. The red circle fits the green curve and the radius of the circle represents the radius of anterior lens curvature.

All the patients were examined by UBM before any laser or surgical treatment was performed and stopped the use of pilocarpine on the day of examination. UBM measurements with a 50-MHz transducer probe (MD-300L; MEDA Co., Ltd., Tianjin, China) were performed for all the patients under dim light in a supine position by an experienced operator. None of the patients was on topical miotics on the day of examination. Scans were centered on the pupil and panoramic images along the horizontal axis (3–9 o'clock) and vertical axis (0–6 o'clock) were used for further analysis. Images with clear visibility of the scleral spurs, more than 3 mm visible continued anterior surface of the lens without motion artifacts were used for analysis of lens curvature. Images were analyzed using Matlab software (Mathworks, Inc. Natick, USA) by a single operator (LTT) who was masked to all the clinical information or the diagnosis at the time of analysis. The only observer input was the identification of two scleral spurs. A horizontal line joining the two scleral spurs was first drawn and then a perpendicular line intersected the middle of the horizontal line. The intersection of the perpendicular line to the anterior surface of lens was defined as the center point and a 3 mm length of the anterior lens surfaces centered on this point was used for calculating ALR. The radius of the surface was calculated by fitting with a circle using a least-squares curve fitting technique [22]. Results from images along the horizontal axis and vertical axis were averaged and taken as the ALR of a certain eye.  Figure 1 displays the key anatomical marks and fitted curves on anterior lens surface (Figure 1).

### 2.3. Statistical Analysis

Statistical analyses were performed using SPSS version 20.0 (SPSS, Inc., Chicago, IL, USA) and R 3.6.1 (http://www.R-project.org). Continuous variables were presented as mean ± standard deviation (SD). Categorical data were presented as percentages. Univariate and multivariate analyses were performed by mixed effect linear models of random effect for repeated measurements of two eyes and post hoc Bonferroni's corrections for multiple comparisons between groups. Non-adjusted model and adjusted model accounting for age, sex, were both used for comparisons of AL, ACD, LT, and ALR. AL was further included in the adjusted model for ALR comparisons. ACD and LT were not included due to its close association with ALR.

## 3. Results

A total of 263 subjects were enrolled, of whom 435 eyes were classified into one of the four groups based on angle configurations under gonioscopy and clinical features: 140 eyes in APAC group, 116 eyes in CPAC group, 84 eyes in PACS group, and 95 eyes in normal control group. 91 eyes were excluded due to severe low vision (lower than 20/200), severe cataract (nuclear opalescence, nuclear color, or cortical grating levels greater than 3 with LOCS III standards), and previous histories of intraocular surgeries or poor image quality. Eyes in PACS group came from three types of patients: 1) fellow eyes of some APAC patients, 2) fellow eyes of some CPAC patients, and 3) both eyes of PACS patients. All PACS eyes must meet the diagnosis criteria described in the Methods section. All the subjects had their IOPs under control at the time of examination. Demographic information and biometric characteristics are summarized in Table 1.

**Figure 2:**
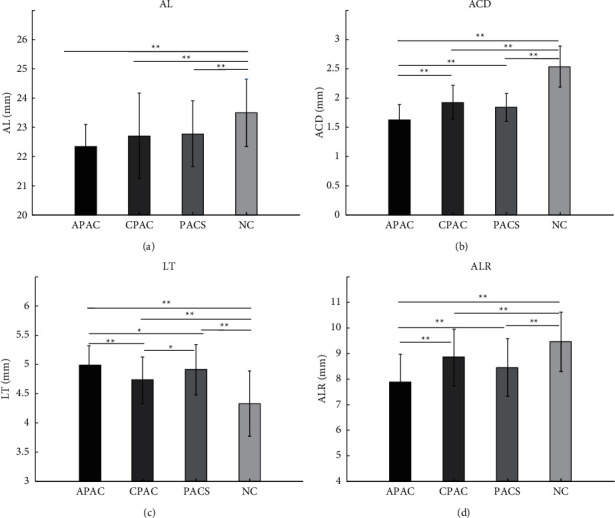
Comparisons of AL (a), ACD (b), LT (c), and ALR (d) measurements between different groups. *P* values are from multivariate analysis with mixed effect linear models adjusting for age and sex. ^*∗∗*^*P* < 0.01. ^*∗*^*P* < 0.05.

Consistent with previous findings, eyes with APAC, CPAC, or PACS tend to have shorter AL (*β* values with 95% CI were −0.91 (−1.24, −0.58), −0.79 (−1.18, −0.40), and −0.81 (−1.17, −0.46) respectively), shallower ACD (*β* values with 95% CI were −0.89 (−0.99, −0.79), −0.65 (−0.75, −0.54), and −0.64 (−0.74, −0.53) respectively), and thicker LT (*β* values with 95% CI were 0.67 (0.53, 0.80), 0.45 (0.31, 0.59), and 0.58 (0.44, 0.72) respectively) than normal eyes after adjusting for age and sex. Furthermore, eyes with APAC have even shallower ACD (*β* values with 95% CI were −0.24 (−0.32, −0.17) and −0.25 (−0.32, −0.19) respectively) and thicker LT (*β* values with 95% CI were 0.21 (0.12, 0.31) and 0.09 (0.02, 0.15) respectively) than eyes with CPAC or PACS. There were no significant differences of AL between APAC and CPAC or PACS. Bar plots of ACD, LT, AL measurements of four groups, and pairwise comparisons between different groups are shown in Figure 2.

ALR is the main outcome measurement in our study, which was 7.90 ± 1.07 mm for APAC eyes, 8.84 ± 1.11 mm for CPAC eyes, 8.45 ± 1.13 mm for PACS eyes, and 9.46 ± 1.16 mm for NC eyes, respectively. Both non-adjusted model and adjusted model accounting for age, sex, and AL were used for comparisons between different groups (Table 2). Overall, there were statistically significant differences of *β* value and 95% CI between group comparisons consistently with either non-adjusted model or adjusted model. Eyes with APAC, CPAC, and PACS all had steeper ALR than normal eyes. ALR in eyes with APAC was even steeper than eyes with CPAC or PACS. There was no statistical difference between eyes with CPAC and PACS.

Among all the subjects that were recruited, 45 subjects had APAC in one eye and PACS in the other eye. Interocular comparison of biometric parameters was made in this subset of subjects (Table 3). When the comparisons were only between APAC eyes and their fellow PACS eyes, there was no statistical difference between AL (22.56 ± 0.73 mm vs. 22.60 ± 1.05 mm, *P* = 0.7573) and LT (5.04 ± 0.37 mm vs. 4.99 ± 0.39 mm, *P* = 0.0997). However, ACD was shallower in APAC eyes than fellow PACS eyes (1.51 ± 0.26 mm vs. 1.76 ± 0.21 mm, *P* < 0.0001). ALR was steeper in APAC eyes than fellow PACS eyes (7.69 ± 1.00 mm vs. 8.11 ± 0.80 mm) as well. Univariate and multivariate analysis with mixed effect linear models were both applied for the interocular comparisons of ALR between APAC and the fellow PACS eyes. *β* value was −0.41 (95% CI = (−0.72, −0.10), *P* value = 0.0096) with the crude model and −0.39 (95% CI = (−0.69, −0.10), *P* value = 0.0094) with the adjusted model accounting for AL, suggesting ALR in APAC eyes was statistically steeper than fellow PACS eyes.

## 4. Discussion

The prevalence of PACG is more significant in China and other Asian countries than worldwide. The understanding of relevant risk factors for PAC and PACG, either epidemiologically, genetically or structurally, can help identify patients with potential risk of angle closure and sometimes provide effective preventive treatment even before the disease onset, such as laser peripheral iridotomy. The mechanisms of primary angle closure can be categorized into pupil-block, anterior nonpupil-block (iris-related, including plateau iris, thick iris roll, decreased light-to-dark change in iris area and anteriorly positioned ciliary processes), lens related (increased thickness and more anteriorly located position), and retrolenticular mechanisms, and these mechanisms can coexist in a single eye [18, 23]. Previous studies have found APAC has more crowded anterior segment and more anteriorly positioned lens as compared to PAC, PACG, or PACS [5–7, 9–13, 15, 24, 25]. A shallow ACD is the most documented and consistent biometric feature predisposing to the acute course of PAC. Other anatomical features related to a more crowded anterior chamber, including angle opening distance (AOD), trabecular-iris space area (TISA), and anterior chamber width, area, and volume (ACW, ACA, ACV) are also associated with APAC. Lens-related anatomical features have been focused on lens thickness, lens position, and lens vault, which is the perpendicular distance from the anterior lens surface to the horizontal line connecting the two scleral spurs and determined by multiple factors, including relative lens position and thickness. APAC eyes tend to have more anteriorly positioned lens with larger lens vault. The forward protruding of the lens occupying more space in AC leads to a more crowded AC. However, whether lens shape, i.e. surface curvature, also contributes to the clinical course of PAC and PACG is not clear.

In this cross-sectional observational study, ALR and other biometric parameters of anterior segment were compared between APAC, CPAC, PACS, and normal eyes. Consistent with previous studies, both acute or chronic PAC and PACS have more crowded anterior segment (shallower ACD, thicker lens) and shorter AL than controls. Furthermore, compared with CPAC and PACS, APAC eyes have even shallower ACD and thicker lens. The relationship of the position and size of lens and the onset of PAC and PACG were commonly studied previously. However, the shape of the lens, especially the anterior surface, has yet to be studied in detail, which may also be an important contributing factor in PAC and PACG. Our previous study using CASIA 2 optical coherence tomography found that steep anterior curvature and decentration of the crystalline lens may be another anatomic characteristic of eyes with PAC/PACG and PACS. [19] Here, we further compared ALR between subgroups of PAC and studied whether it also contributes to the clinical course of PAC and PACG based on UBM imaging. UBM imaging was used in this study because UBM has the advantage to view zonular status and ciliary structure and we routinely perform UBM in PAC/PACS patients to identify whether there would be certain secondary conditions. There have been a few studies that have used UBM to study lens curvature with accommodative changes. [26] We found that the anterior lens surface was steeper in APAC eyes than CPAC, PACS, and controls. This indicated that ALR is not only an anatomical feature related to PAC and PACG, but also associated with the clinical course of the disease. To the best of our knowledge, no comparison on lens curvature between these conditions has been studied before.

The anterior surface of the lens is an important part of the iris-lens channel that determines the resistance of aqueous flow from the poster chamber to the anterior chamber. The tightness and area of iridolenticular apposition would affect the resistance and predispose PAC. Pupillary block and lens related mechanisms are considered to be more relevant in APAC. The relative position between the anterior surface of the lens and the posterior surface of iris near the pupil can cause a pressure gradient between posterior chamber and anterior chamber, and hence, contribute to the angle blocking mechanism. Steeper ALR combined with shallow ACD and tight iridolenticular contact may increase the pressure gradient due to an increased resistance of aqueous flow, thus results in pupillary block.

The difference of anatomical features of the lens may be determined by physiological differences of lens per se among eyes. Another explanation could be physiological differences of other structures that act on the lens. Uveal expansion has been considered as one possible contributing factor. [27] Expansion of choroid could increase the pressure in vitreous body and cause anterior rotation of ciliary body, which could affect lens structure and position. The stability of zonules is another factor that may affect lens structure. Loose zonules could make the lens more mobile and often repositioned anteriorly. The lens zonulysis not only changes the position of the lens, but may also change the curvature of lens surface. Studies on accommodation influence in normal subjects showed that lens surface curvature increases during accommodation, although the ability of deformation reduces as aging [26, 28, 29]. Also, loose zonules are a feature of exfoliation syndrome, and there are evidences indicating this condition has a higher prevalence of PACG [30]. This also supports the hypothesis that we propose, which is zonule tension may be more loose in APAC eyes which affects the lens curvature and predisposes acute attack. Thus, we suggest that the differences of ALR between APAC, CPAC, and PACS eyes may be partially related to zonule tension differences as crystalline lens dislocation was often seen in PACG eyes [19, 31, 32].

Confounding issues that may affect our results include whether the acute attack has resolved, the use of pilocarpine, and the severity of cataract. All of our patients had resolved from acute attack before recruitment. In PAC and PACG subjects, the use of pilocarpine can cause change of accommodation state and possible change of zonule tension. Here in our study, all eyes diagnosed with APAC or CPAC and most of the eyes diagnosed with PACS were prescribed with 0.5% pilocarpine upon diagnosis and stopped use on the day of exam. Early study found that the effect of 2% pilocarpine on anterior chamber depth and lens thickness was maximal 45 to 60 minutes after instillation, and virtually gone after 100 minutes [33]. Since the concentration of pilocarpine in our study was lower than that commonly used in the literature, we stopped the use of pilocarpine on the day of exam which is sufficient based on previous literature, and since the percentage of pilocarpine usage was generally comparable among APAC, CPAC, and PACS groups, we believe the effect of pilocarpine was minimal and does not affect our main conclusion. The severity of cataract, especially in intumescent stage, may also affect the lens curvature. We have excluded patients with severe cataract based on LOCS III standards (nuclear opalescence, nuclear color, or cortical gratings greater than 3) and UBM reflection, to minimize the effect of cataract on lens curvature.

## 5. Conclusion

PACG is more prevalent in China and other Asian countries and has a more devastating effect on visual function, especially the acute type. Finding anatomical factors that may affect the clinical course of PACG will provide further insight into the mechanism of angle closure glaucoma and clinical management. In addition to a more crowded anterior segment in APAC than CPAC, PACS, and normal controls, we also found that ALR is steepest in APAC, indicating that ALR is also an important anatomical feature that contributes to the clinical course of PACG. The differences of ALR among subtypes of PAC could be due to the physiological difference of lens per se, or zonular tension might be a possible factor that acts on the lens and changes its shape and position.

## Figures and Tables

**Table 1:** Comparison of demographic data and biometric characteristics of APAC, CPAC, PACS, and normal control groups.

Characteristics	APAC	CPAC	PACS	NC	*P* value

By subject	*N* = 111	*N* = 78	*N* = 75	*N* = 54	
Age (yrs)	66.05 ± 8.49	63.36 ± 10.32	67.37 ± 8.35	66.70 ± 8.96	0.036
*Gender (%)*					<0.001
Male	24 (21.62)	36 (46.15)	15 (20.00)	19 (35.19)	
Female	87 (78.38)	42 (53.85)	60 (80.00)	35 (64.81)	

By eye	*N* = 140	*N* = 116	*N* = 84	*N* = 95	
AL (mm)	22.34 ± 0.76	22.71 ± 1.46	22.78 ± 1.13	23.50 ± 1.15	<0.001
ACD (mm)	1.63 ± 0.26	1.93 ± 0.29	1.84 ± 0.24	2.54 ± 0.35	<0.001
LT (mm)	4.99 ± 0.33	4.73 ± 0.40	4.91 ± 0.43	4.33 ± 0.56	<0.001
Kf (D)	44.24 ± 1.53	44.20 ± 1.54	43.86 ± 1.59	43.72 ± 1.99	0.055
Ks (D)	45.29 ± 1.76	45.15 ± 1.66	44.87 ± 1.97	44.33 ± 2.06	<0.001
ALR (mm)	7.90 ± 1.07	8.84 ± 1.11	8.45 ± 1.13	9.46 ± 1.16	<0.001

Continuous values are presented as mean ± standard deviation (SD). Categorical data are presented as percentage. *P* values are from univariate analysis with mixed effect linear models. APAC, acute primary angle closure; CPAC, chronic primary angle closure; PACS, primary angle closure suspect; NC, normal controls; AL, axial length; ACD, anterior chamber depth; LT, lens thickness; Kf, flat keratometry; Ks, steep keratometry; ALR, radius of anterior lens curvature.

**Table 2:** Univariate and multivariate analysis with mixed effect linear models for ALR comparisons between different groups.

Groups	Crude model		Adjusted model for age, sex		Adjusted model for age, sex, and AL	
	*β* value (95%CI)	*P*	*β* value (95%CI)	*P*	*β* value (95%CI)	*P*
APAC vs. NC	−1.55 (−1.91, −1.19)	<0.0001	−1.55 (−1.91, −1.18)	<0.0001	−1.37 (−1.78, −0.97)	<0.0001
CPAC vs. NC	−0.69 (−1.08, −0.31)	0.0004	−0.69 (−1.08, −0.30)	0.0005	−0.60 (−1.03, −0.17)	0.006
PACS vs. NC	−1.03 (−1.42, −0.63)	<0.0001	−1.02 (−1.42, −0.63)	<0.0001	−0.93 (−1.34, −0.52)	<0.0001
APAC vs. CPAC	−0.86 (−1.18, −0.54)	<0.0001	−0.86 (−1.19, −0.52)	<0.0001	−0.78 (−1.11, −0.44)	<0.0001
APAC vs. PACS	−0.53 (−0.81, −0.24)	0.0004	−0.52 (−0.81, −0.23)	0.0004	−0.45 (−0.74, −0.16)	0.002
CPAC vs. PACS	0.33 (−0.01, 0.68)	0.056	0.33 (−0.02, 0.69)	0.066	0.33 (−0.03, 0.69)	0.075

APAC, acute primary angle closure; CPAC, chronic primary angle closure; PACS, primary angle closure suspect; NC, normal controls; *β* values (95%CI) and *P* values are from univariate (crude model) and multivariate (adjusted model) analyses with mixed effect linear models. The statistical significances are consistent between different models.

**Table 3:** Interocular comparison of biometric parameters in APAC eyes and fellow PACS eyes.

	AL (mm)	ACD (mm)	LT (mm)	ALR (mm)
APAC (Mean ± SD) *N* = 45	22.56 ± 0.73	1.51 ± 0.26	5.04 ± 0.37	7.69 ± 1.00
PACS (Mean ± SD) *N* = 45	22.60 ± 1.05	1.76 ± 0.21	4.99 ± 0.39	8.11 ± 0.80
*β* value (95% CI)	−0.04 (−0.26, 0.19)	−0.25 (−0.33, −0.17)	0.06 (−0.01, 0.13)	−0.41 (−0.72, −0.10)
*P* value	0.7573	<0.0001	0.0997	0.0096

APAC, acute primary angle closure; CPAC, chronic primary angle closure; PACS, primary angle closure suspect; NC, normal controls; *β* values (95%CI) and *P* values are from univariate analyses with mixed effect linear models.

## Data Availability

The data used to support the findings of this study are presented in the tables. The Matlab scripts for calculations with UBM images are available from the corresponding author on reasonable request.
